# Central Sensitization Is Associated with Inferior Patient-Reported Outcomes and Increased Osteotomy Site Pain in Patients Undergoing Medial Opening-Wedge High Tibial Osteotomy

**DOI:** 10.3390/medicina58121752

**Published:** 2022-11-29

**Authors:** Jae-Jung Kim, In-Jun Koh, Man-Soo Kim, Keun-Young Choi, Ki-Ho Kang, Yong In

**Affiliations:** 1 Department of Orthopaedic Surgery, Seoul St. Mary’s Hospital, College of Medicine, The Catholic University of Korea, 222, Banpo-daero, Seocho-gu, Seoul 06591, Republic of Korea; 2 Department of Orthopaedic Surgery, EunPyeong St. Mary’s Hospital, College of Medicine, The Catholic University of Korea, 1021, Tongil Ro, Eunpyeong-gu, Seoul 03312, Republic of Korea

**Keywords:** medial opening-wedge high tibial osteotomy, central sensitization, patient-reported outcomes, osteotomy site pain, minimal clinically important difference

## Abstract

*Background and Objectives*: Studies have shown that centrally sensitized patients have worse clinical outcomes following total knee arthroplasty (TKA) than non-centrally sensitized patients. It is unclear whether central sensitization (CS) affects patient-reported outcomes (PROs) and/or level of osteotomy site pain in patients undergoing medial opening-wedge high tibial osteotomy (MOWHTO). The purpose of this study was to determine whether CS is associated with PROs and osteotomy site pain following MOWHTO. *Materials and Methods*: A retrospective evaluation was conducted on 140 patients with varus knee osteoarthritis (OA) who were treated with MOWHTO and monitored for two years. Before surgery, the Central Sensitization Inventory (CSI) was used to assess CS status, and a CSI of 40 or higher was considered indicative of CS. The Western Ontario and McMaster Universities Osteoarthritis Index (WOMAC) and pain visual analogue scale (VAS) were used to assess PROs. The minimal clinically important difference (MCID) for the WOMAC was set as 4.2 for the pain subscore, 1.9 for the stiffness subscore, 10.1 for the function subscore, and 16.1 for the total based on the results of a previous study. The WOMAC score, pain VAS score of the osteotomy site, and the achievement rates of WOMAC MCID were compared between the CS and non-CS groups. *Results*: Thirty-seven patients were assigned to the CS group, whereas 84 were assigned to the non-CS group. Before surgery, the CS group showed a higher WOMAC score than the non-CS group (58.7 vs. 49.4, *p* < 0.05). While there was a statistically significant improvement in WOMAC subscores (pain, stiffness, function, and total) for both groups at two years after surgery (all *p* < 0.05), the CS group had a higher WOMAC score than the non-CS group (37.1 vs. 21.8, *p* < 0.05). The CS group showed significantly inferior results in pre- and postoperative changes of WOMAC subscores (pain, function, and total) relative to the non-CS group (all *p* < 0.05). In addition, pain at the osteotomy site was more severe in the CS group than in the non-CS group at two years after surgery (4.8 vs. 2.2, *p* < 0.05). Patients with CS had worse MCID achievement rates across the board for WOMAC pain, function, and total scores (all *p* < 0.05) compared to the non-CS group. *Conclusions*: Centrally sensitized patients following MOWHTO had worse PROs and more severe osteotomy site pain compared to non-centrally sensitized patients. Furthermore, the WOMAC MCID achievement rate of patients with CS was lower than that of patients without CS. Therefore, appropriate preoperative counseling and perioperative pain management are necessary for patients with CS undergoing MOWHTO. *Level of Evidence*: Level III, case-control study.

## 1. Introduction

Medial compartment osteoarthritis (OA) with varus deformity [1,2] is the most common indication for medial opening-wedge high tibial osteotomy (MOWHTO). Chondral lesions in the medial compartment [3], osteonecrosis of the medial femoral condyle [4], and varus thrust instability [3] are all reasons to consider MOWHTO if they are accompanied by varus deformity. By moving the mechanical axis to the lateral compartment, it is possible to reduce load on the medial compartment and postpone the progression of knee OA [5,6].

While total knee arthroplasty (TKA) is effective in eliminating pain by cutting off the source at the joint periphery, about 20–40% of patients are dissatisfied with the results and complain of persistent pain [7]. Due to these results, research into the mechanisms of central pain has increased [8]. Studies have shown that centrally sensitized patients had worse clinical outcomes and pain following TKA than non-centrally sensitized patients [9,10,11]. Central sensitization (CS) is considered an etiology of persistent pain and dissatisfaction following TKA [9,10,11,12]. Hyperalgesia and allodynia are hallmarks of CS, which is caused by dysfunction in the central nervous system [10,11]. However, there is a lack of studies on patient-reported outcomes (PROs) in CS patients following MOWHTO. In addition, unlike TKA, in which the painful lesion is surgically removed, MOWHTO is performed on the bone around the lesion. Currently, no studies have examined the PROs and degree of osteotomy site pain after MOWHTO according to the presence or absence of CS.

PROs have gained traction in recent years, and patient-reported outcome measures (PROMs) are widely used to assess the success of orthopedic procedures [13]. One of the most common and reliable PROMs used following knee surgery is the Western Ontario and McMaster Universities Arthritis Index (WOMAC) [14,15]. The pain visual analogue scale (VAS) score is also extensively used for evaluating outcomes following orthopedic surgery [16]. Understanding the minimal clinically important difference (MCID) and factors that may be used to forecast whether the MCID will be exceeded might have a major bearing on the definition of surgical outcomes and on the development of patient-centered decision-making aids [17,18]. Due to its emphasis on clinical relevance over statistical significance, it is recommended to use the MCID in PROMs [18,19].

It is currently unclear whether CS affects PROs and/or osteotomy site pain in patients undergoing MOWHTO. Therefore, the purpose of this study was to determine whether CS is associated with PROs and osteotomy site pain following MOWHTO. We predicted that centrally sensitized patients would have worse PROs and increased osteotomy site pain than those who were not centrally sensitized.

## 2. Materials and Methods

**Figure 1:**
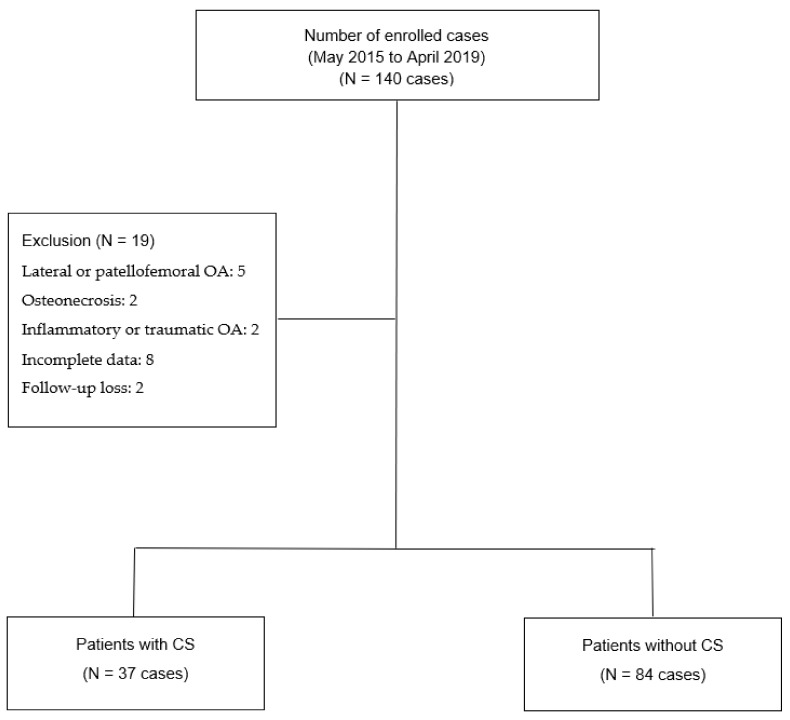
Patient flowchart.

As part of a retrospective record review research, we included 140 patients who received MOWHTO between May 2015 and April 2019 at a single hospital. The criteria for inclusion were age < 70 years and isolated medial compartment OA with varus deformity. Patients with lateral compartment and patellofemoral OA, osteonecrosis, inflammatory or traumatic OA, missing data, and/or loss to follow-up within two years were excluded from the study to reduce the potential impact of preoperative variables on MOWHTO outcomes. The final study contained 121 patients, after excluding 19 for various reasons (Figure 1). Informed consent was acquired from all patients, and the study was approved by the Institutional Review Board of Seoul St. Mary’s Hospital (KC22RASI0419 and 15 June 2022).

*Surgical procedure*

A tourniquet was placed in the proximal femur and maintained during surgery with a pressure of 300 mmHg. Arthroscopy was performed prior to osteotomy in all cases. If the meniscus tear was present including medial meniscal root tear, partial meniscectomy was performed. Multiple drilling was also performed for cartilage defects. A 7 cm long skin incision was made in the middle between the tibial tuberosity and the posteromedial border of the tibia, beginning 1 cm below the knee joint. Same skin incision was used in all patients. The Dugdale method [20] was used to determine the correction angle, and the Fujisawa point [21] was chosen as the surgical target. The Dugdale method requires the use of two lines to calculate the correction angle. Two lines are drawn: one from the center of the femoral head to 62.5% of the tibial width and the other from the center of the tibiotalar joint to the same percentage of the tibial width. The angle between these two lines is known as the correction angle [22]. Biplanar osteotomy was used in the surgical operation. A locking plating system (TomoFix^®^, DePuy Synthes, Oberdorf, Switzerland) was used to secure the osteotomy site. No bone grafts or artificial materials were used to fill the opening gap space.

*Postoperative rehabilitation*

Following surgical procedures, all patients participated in the same rehabilitation regimen. To improve knee flexion range, exercise on a continuous passive motion machine was started on postoperative day one at 60 degrees and progressed by 5 degrees daily up to 130 degrees of flexion. Within four weeks, patients were allowed to walk with crutches, and after six weeks, they were permitted full weight bearing.

*Clinical assessment and Radiographic evaluation*

The Central Sensitization Inventory (CSI) was used to assess the status of CS, and patients were given the questionnaire the day before the surgery [23]. The CSI is a valid and reliable self-report assessment of central sensitivity [23,24]. The purpose of this 25-item survey is to evaluate both emotional state and pain perception in the course of daily living. Each survey uses a Likert scale ranging from 0 to 4 to collect data. Based on the findings of prior research, a CSI of 40 or higher is considered indicative of CS, and a CSI score less than 40 indicates non-CS [24]. In this study, 37 patients were assigned to the CS group, whereas 84 were assigned to the non-CS group (Figure 1).

Preoperatively and at two years postoperatively, the WOMAC scores were used to assess knee PROs [25]. The 24-item disease-specific questionnaire (WOMAC) is a popular tool for assessing patient progress following knee surgery. It is comprised of three subscores evaluating pain (five items), stiffness (two items), and function (17 items). Each of the items has five possible responses, with scores ranging from 0 to 4. Scores range from 0 to 96, with 20 points assigned to pain, 8 points to stiffness, and 68 points to function. A lower score indicates a more favorable clinical result. In addition, pain VAS scores were used to assess pain at the osteotomy site at two years after surgery. The MCID for the WOMAC was set as 4.2 for the pain subscore, 1.9 for the stiffness subscore, 10.1 for the function subscore, and 16.1 for the total score based on the results of a previous study [26] that refers to the smallest improvement that a patient might perceive as beneficial. The achievement rates of WOMAC MCID were also measured. Between the CS and non-CS groups, the WOMAC scores, pain VAS scores of the osteotomy site, and the achievement rates of WOMAC MCID were compared.

Age, sex, operation side, body mass index (BMI), American Society of Anesthesiologists (ASA) score, comorbidities (diabetes and hypertension), alcohol intake, smoking, and preoperative OA severity were also recorded. The Kellgren and Lawrence (K-L) grade was used to quantify the severity of OA [27]. Hip–knee–ankle (HKA) angle and weight-bearing line (WBL) ratio were assessed as radiological variables preoperatively and after 2 years postoperatively. HKA angle is a measure of lower limb alignment, defined as the angle between the mechanical axes of the femur and the tibia. The WBL ratio was estimated by measuring from the medial border of the proximal tibia to the spot where the WBL meets the proximal tibia and dividing that number by the total width of the tibia. HKA angle and WBL ratio were measured on weight-bearing bilateral standing long-leg radiographs. In addition, correction angles were collected for surgical factors.

Preoperative and postoperative HKA angle and WBL ratio were analyzed twice by two orthopedic surgeons. They were orthopedic surgeons with more than five years of experience. Each analyzer was blinded to the results of the other and to all patient information. The average of the two analyzers’ results was used. Using the intraclass correlation coefficient (ICC), intraobserver and interobserver reliability were evaluated to determine the reliability of the measurement. In this study, both intraobserver and interobserver ICC values were greater than 0.8.

*Statistical Analysis*

The mean and standard deviation are presented as descriptive statistics for all continuous variables, and frequency statistics are supplied for all noncontinuous variables. We compared preoperative PROMs, two-year postoperative PROMs, and osteotomy site pain between the CS and non-CS groups using Student’s *t*-test. The rate of WOMAC MCID achievement was compared between the two groups using the chi-square test. A post hoc power analysis was conducted to evaluate whether our findings had sufficient statistical power. SPSS was used conduct statistical analyses (version 21.0; IBM). A *p*-value less than 0.05 was considered statistically significant.

## 3. Results

The 121 patients included 37 (30.5%) patients with CS and 84 (69.4%) patients without CS. The mean follow-up period was 2.0 ± 0.1 years. Table 1 shows the demographic data comparing patients with and without CS.

Before surgery, the CS group showed a significantly higher mean WOMAC score than the non-CS group (*p* < 0.05, Table 2). While there was a statistically significant improvement in WOMAC subscores (pain, stiffness, function, and total) for both groups (all *p* < 0.05), the CS group had a significantly lower two-year postoperative score and lower WOMAC subscores (pain, function, and total) than in the non-CS group (all *p* < 0.05, Table 2). In addition, pain VAS at the osteotomy site was more severe in the CS group at two years after surgery (*p* < 0.05, Table 2). With an alpha value of 0.05, a post hoc power analysis revealed greater than 90% power to detect a difference in postoperative two-year WOMAC (pain, function, and total) and postoperative two-year osteotomy site pain VAS.

MCID achievement was examined using the results of a previous study [26]. Patients with CS had worse MCID achievement rates across the board for WOMAC pain, function, and total scores (all *p* < 0.05) compared with the non-CS group (Table 3).

During the follow-up, there were no major complications requiring additional surgery. Minor complications occurred in four knees. Three patients had superficial wound infections that were treated with intravenous antibiotics. Routine DVT evaluation was not performed. However, one symptomatic patient was confirmed as DVT under CT venography and was treated with oral anticoagulants. No neurovascular injury, pulmonary embolism, or delayed union case was observed during the follow-up period.

## 4. Discussion

The most important finding of this study was that patients with CS showed inferior outcomes in terms of PROs and greater osteotomy site pain compared to those without CS despite significant improvement in WOMAC subscores.

It has been established that CS is a major contributor to persistent pain following TKA [9,10,11,12]. It is believed that severe chronic pain is induced by reduced activation of the descending antinociceptive pathway, perhaps as a result of inactivation by norepinephrine/serotonin [10,11]. Unlike neurogenic or inflammatory pain, central pain is defined as dysfunctional [28,29]. The Central Sensitization Inventory (CSI) is frequently used to evaluate CS [23,24]; in addition, a whole-body pain diagram or quantitative sensory testing (QST) can be used for assessment [30,31,32,33]. In our study, the CSI was used for diagnosis of CS.

There have been many studies that focused on CS in relation to the spine and joints, especially in the field of TKA. It has been reported that CS patients showed inferior PROs to non-CS patients in the short term (three months to two years) after TKA [9,10,11].

Although MOWHTO is a surgical procedure that also involves bone cutting, there has been no research on the relationships between clinical outcomes and CS. As such, this study investigated the relationships between CS and clinical outcomes following MOWHTO.

Even though the PROs of the CS group showed significant improvement after surgery, scores for the preoperative, postoperative, and amount of change in WOMAC in patients with CS were worse than those for patients without CS. Two years after surgery, pain at the osteotomy site of patients with CS was more severe than in non-CS patients. Therefore, surgery outcome, including osteotomy site pain, may be inferior for CS patients who undergo MOWHTO.

Most recently, the MCID (minimum gain judged clinically important vs. statistically significant) has been given more weight [34,35]. Due to the lower improvement in WOMAC scores of patients with CS compared to those without CS, obtaining a minimally acceptable improvement in clinical outcomes is more difficult. In our study, the WOMAC scores of CS patients improved statistically significantly after surgery, but the WOMAC MCID achievement rate of the CS group was only 56.8%. This means that it is difficult to expect clinical improvement in nearly half of patients when performing MOWHTO in the CS group, possibly explaining why CS patients who undergo MOWHTO report lower levels of satisfaction.

Although there was no statistically significant difference in sex between the two groups, the proportion of females in the CS group tended to be higher than in the non-CS group (*p* = 0.064). Women have been shown in several studies to be more sensitive to centrally mediated pain than males [36,37,38]. This is due to their decreased modulation of conditioned pain [36], increased referred pain following intramuscular experimental pain [37], and increased temporal summation to thermal pain [38]. However, there has been a lack of studies that clearly examine sex differences in pain sensitivity in patients with knee OA, which may be attributable to the confounding factor of disease.

The reason for the differences in PROs and osteotomy site pain between patients with and without CS is not well understood. It is considered that individuals with and without CS have different clinical results after surgery [9,10,11]. Even before surgery, patients with CS had worse pain and function than patients without CS. Despite the clinical improvement following surgery for both groups, patients with CS might experience more pain in response to the same stimulus (hyperalgesia) and could interpret normal stimulation as painful (allodynia) [9,10]. As a result, it is recommended that patients with CS be given different perioperative pain control when assessing the PROs and osteotomy site pain in patients undergoing MOWHTO. With an incidence of 30.5% of patients with CS in our study, active pain control regimens should be incorporated focusing on preemptive, multimodal pain management in patients with CS undergoing MOWHTO. Studies exist that have demonstrated the efficacy of duloxetine in TKA patients [11,39,40]. Additionally, a recent study suggested that perioperative duloxetine could reduce pain after HTO [41]. Although we think duloxetine can help control pain in MOWHTO patients, further randomized study is warranted. A better understanding of differences in PROs and osteotomy site pain between patients with and without CS for MOWHTO would lead to better overall patient perioperative pain management.

There were limitations in this study. First, as seen in previous research with Koreans who have been diagnosed with OA, patients in this study were all Korean, with females as the majority of patients, which limits the application of the study to other environments [42,43]. In Korea, women have been shown to account for 80–90% of cases of degenerative arthritis and TKA [44,45], indicating an acceptable degree of bias in our study. Second, as a retrospective analysis, there is a chance of selection bias, even though CSI and WOMAC scores were prospectively assessed in all patients. Additional prospective studies are required to verify these findings. Third, due to the nature of the study, only 37 patients were included in the CS group, even though 121 patients who had MOWHTO were studied to compare the PROs and osteotomy site pain according to CS [46,47]. However, our preoperative prevalence of CS was consistent with other studies that found central sensitization in 20–40% of patients before surgery [46,47,48,49], which was 30.5% in our study. Fourth, there is a variety of questionnaires that can be used to evaluate the degree of CS [23,24,30,31,32,33], which means that study results may vary depending on which questionnaire is used. Unfortunately, there is no agreed-upon approach for assessing CS, and it is unclear whether previous evaluations were validated for use in knee OA patients undergoing MOWHTO. In this study, CS was evaluated using the CSI, which should be considered before applying our results to other studies. Finally, to determine the degree of WOMAC score improvement, we depended on the previously reported MCID of 16.1 points [26]. It has been reported that there are several variables related to MCID, and these differences are clinically significant [34,35]. Despite these limitations, the findings in this study are consistent with those of previous studies, indicating some degree of generalizability [11,46,47]. Therefore, this study contributes important knowledge on CS and its impact on PROs following MOWHTO.

## 5. Conclusions

The PROs and severity of osteotomy site pain of patients with CS following MOWHTO were worse than those of patients without CS. Moreover, the MCID achievement rate of patients with CS was lower than that of patients without CS. Therefore, appropriate preoperative counseling and perioperative pain management should be considered for patients with CS undergoing MOWHTO.

## Figures and Tables

**Table 1:** Patient characteristics.

Variables	CS Group (*n* = 37)	Non-CS Group (*n* = 84)	*p* Value
Age (years)	57.6 ± 5.8	55.9 ± 6.1	0.170
Sex			0.064
Male	1 (2.7%)	12 (14.3%)	
Female	36(97.3%)	72 (85.7%)	
Operation side			0.891
Right	18 (48.6%)	42 (50.0%)	
Left	19 (51.4%)	42 (50.0%)	
Body mass index (kg/m^2^)	26.1 ± 4.1	26.2 ± 3.1	0.817
CSI score	45.3 ± 6.7	21.2 ± 8.6	0.000
K-L grade			0.415
2	11 (29.7%)	17 (20.2%)	
3	25 (67.5%)	61 (72.6%)	
4	1 (2.7%)	6 (7.1%)	
Preoperative HKA angle, deg	6.9 ± 2.9	7.2 ± 2.8	0.529
Preoperative WBL ratio, %	19.3 ± 11.9	16.8 ± 11.3	0.267
Postoperative two-year HKA angle, deg	−1.4 ± 2.0	−0.9 ± 2.5	0.277
Postoperative two-year WBL ratio, %	55.1 ± 10.9	53.5 ± 9.8	0.447
Surgical correction angle	10.5 ± 2.7	11.2 ± 2.7	0.243
ASA score			0.275
1	15 (40.5%)	23 (27.3%)	
2	22 (59.4%)	59 (70.2%)	
3	0 (0.0%)	2 (2.3%)	
Diabetes	5 (13.5%)	7 (8.3%)	0.510
Hypertension	12 (32.4%)	24 (28.6%)	0.669
Alcohol consumption	6 (16.2%)	16 (19.0%)	0.710
Smoking	5 (13.5%)	8 (9.5%)	0.534

Data are provided as mean ± SD or *n* (%). CS, central sensitization; CSI, Central Sensitization Inventory; K–L, Kellgren–Lawrence; ASA, American Society of Anesthesiologists.

**Table 2:** Preoperative and two-year postoperative WOMAC scores and two-year postoperative osteotomy site pain.

Variables	CS Group (*n* = 37)	Non-CS Group (*n* = 84)	*p* Value
Preoperative WOMAC			
Pain	11.8 ± 3.0	9.3 ± 3.3	0.000
Stiffness	5.0 ± 1.7	4.4 ± 2.2	0.198
Function	41.8 ± 9.3	35.6 ± 11.7	0.003
Total	58.7 ± 12.5	49.4 ± 16.1	0.001
Postoperative two-year WOMAC			
Pain	7.8 ± 2.2	3.9 ± 3.5	0.000
Stiffness	2.7 ± 1.1	2.2 ± 1.6	0.067
Function	26.5 ± 4.8	15.6 ± 11.3	0.000
Total	37.1 ± 6.2	21.8 ± 14.6	0.000
Change WOMAC			
Pain	3.9 ± 2.1	5.3 ± 3.0	0.016
Stiffness	2.3 ± 1.6	2.2 ± 2.1	0.897
Function	15.3 ± 8.7	19.9 ± 14.3	0.031
Total	21.5 ± 9.7	27.5 ± 17.6	0.019
Postoperative two-year osteotomy site pain VAS	4.8 ± 1.8	2.2 ± 2.1	0.000

Data are provided as mean ± SD or *n* (%). WOMAC, Western Ontario and McMaster Universities arthritis index score; VAS, visual analogue scale.

**Table 3:** MCID achievement.

Variables	CS Group (*n* = 37)	Non-CS Group (*n* = 84)	*p* Value
MCID achievement			
Pain	23 (62.2%)	67 (79.8%)	0.041
Stiffness	19 (51.4%)	44 (52.4%)	0.917
Function	21 (56.8%)	64 (76.2%)	0.031
Total	21 (56.8%)	64 (76.2%)	0.031

Data are provided as mean ± SD or *n* (%). MCID, minimal clinically important difference.

## Data Availability

The data published in this research are available on request from the first author (J.-J.K).
